# Equity of inpatient health care in rural Tanzania: a population- and facility-based survey

**DOI:** 10.1186/1475-9276-11-7

**Published:** 2012-02-15

**Authors:** Grace A Ferry, Sean R Dickson, Godfrey Mbaruku, Lynn P Freedman, Margaret E Kruk

**Affiliations:** 1 Mathematica Policy Research 1100 1st Street Northeast Washington, DC 20011 USA; 2 Department of Health Management and Policy University of Michigan School of Public Health 1415 Washington Heights Ann Arbor, MI 48109 USA; 3 Ifakara Health Institute Plot 463, Kiko Avenue Mikocheni P.O. Box 78 373 Dar es Salaam Tanzania; 4 Department of Population and Family Health Columbia University Mailman School of Public Health 60 Haven Avenue New York, NY 10032 USA; 5 Department of Health Policy and Management Columbia University Mailman School of Public Health 600 West 168th Street New York, NY 10032 USA

## Abstract

**Objective:**

To explore the equity of utilization of inpatient health care at rural Tanzanian health centers through the use of a short wealth questionnaire.

**Methods:**

Patients admitted to four rural health centers in the Kigoma Region of Tanzania from May 2008 to May 2009 were surveyed about their illness, asset ownership and demographics. Principal component analysis was used to compare the wealth of the inpatients to the wealth of the region's general population, using data from a previous population-based survey.

**Results:**

Among inpatients, 15.3% were characterized as the most poor, 19.6% were characterized as very poor, 16.5% were characterized as poor, 18.9% were characterized as less poor, and 29.7% were characterized as the least poor. The wealth distribution of all inpatients (p < 0.0001), obstetric inpatients (p < 0.0001), other inpatients (p < 0.0001), and fee-exempt inpatients (p < 0.001) were significantly different than the wealth distribution in the community population, with poorer patients underrepresented among inpatients. The wealth distribution of pediatric inpatients (p = 0.2242) did not significantly differ from the population at large.

**Conclusion:**

The findings indicated that while current Tanzanian health financing policies may have improved access to health care for children under five, additional policies are needed to further close the equity gap, especially for obstetric inpatients.

## Introduction

Although government expenditures for health in low- and middle-income countries are intended to ensure access to health care for the poor, they frequently disproportionately benefit wealthier citizens [1,2]. A review of public expenditures on health in 26 developing countries found that the poorest fifth of a population typically received less than a fifth of government expenditures on health [3]. Another study published by O'Donnell and colleagues found that the concentration index of public health spending--a measure of the wealth distribution of spending--in Asia was pro-rich in 7 of the 11 countries/regions studied: Nepal, Bangladesh, Indonesia, India, Vietnam, and Gansu and Heilongjiang provinces in China [4]. The most pro-rich financing was found in Nepal, the poorest country.

User fees, or out-of-pocket payments, at the point of care are used extensively to raise revenues in many poor countries. Researchers have established that user fees contribute to inequities by decreasing the use of needed health services by the poor and near-poor [5-7]. Informal or illegal fees and other costs, such as transportation expenses, also contribute to inequities by making health care unaffordable for poorer individuals [8-11].

Several studies have indicated that removing user fees can reduce, although not eliminate, barriers to access [12,13]. For example, a study in Ghana found that while removing user fees for deliveries helped to narrow the equity gap for deliveries at facilities, other costs--such as transportation expenses--remained substantial and meant that the wealthy were more likely to deliver in a health facility than their poorer counterparts [14].

Tanzania, a low-income country in East Africa, removed user fees for maternal and child (under-5) health services, as well as fees for individuals over age 60 and those with specific medical conditions (e.g, HIV, TB) as part of an effort to promote equitable utilization of essential health care. While there is evidence that the exemptions have not eliminated wealth-based disparities in access to outpatient and obstetric delivery services, there is less information about the equity of inpatient services [10,15,16]. As inpatient services address more serious conditions, inequitable utilization across socioeconomic status may have important health consequences.

One approach to assessing equity of inpatient admissions is a wealth comparison between inpatients and the general population. This has been done by researchers in Bangladesh who demonstrated that a simplified wealth asset patient questionnaire can be applied in a low-income hospital to study equity in access to emergency obstetric care [17].

The objective of this study was to explore the equity of utilization of maternal, child, and other adult inpatient health care in Tanzania. We used a short admission questionnaire that contained information on household assets--a measure of permanent income--and compared the wealth distribution of inpatients to that of the surrounding communities, using data from a previous population-based survey. We were particularly interested in comparing equity of utilization of fee-exempt and non fee-exempt services. We also wanted to qualitatively assess the feasibility of such an approach for ongoing monitoring of equitable health service provision in a low-income country. To our knowledge, this is the first application of this approach in Africa.

## Methods

### Study setting

Data was collected at four rural health centers (Bitale, Nguruka, Kakonko, and Mabamba) in Tanzania's Kigoma Region, a western region bordering Burundi and separated from the Democratic Republic of Congo by Lake Tanganyika. Bitale and Nguruka are located in the Kigoma Rural district and Kakonko and Mabamba are located in Kibondo district. Nguruka, Kakonko, and Mabamba are all receiving new staff houses and operating theaters as part of health facility upgrades. Facility and patient level data were collected for four months, May 2008, September 2008, January 2009, and May 2009. The data collected is part of a larger study to prospectively assess the impact of quality upgrades in three health centers (plus one comparison health center without upgrades) on overall maternal health care utilization in the Kigoma Region of Tanzania.

The health centers provide both primary and secondary care. The user fee to receive inpatient services was 2,000 TZS or 1.50 USD. In 2002, the gross national income per capita was $290 [18]. User fee exemptions are provided to the following: individuals under the age of five or over the age of sixty, pregnant mothers (e.g., deliveries, antenatal care, and postnatal care), and individuals with exempt medical conditions (e.g., HIV/AIDS, tuberculosis, diabetes, and cancer) [19]. The health centers also accept national health insurance, health benefits for government employees, and community fund insurance, a national prospective payment program that costs 5,000 TZS or 3.75 USD per year and covers services for an individual and their immediate family at dispensaries and health centers (catastrophic expenses are excluded) [19].

### Facility-level data

Project managers collected facility-level data at the beginning and end of each monthly data collection period. Facility-level data tracked included facility inputs (e.g., staffing levels, functionality of equipment, training courses offered, and progress on health center upgrades) and facility outputs (e.g., total admissions and length of stay).

### Patient level data collection

The patient survey and consent form were developed in English, translated into Swahili, and then back translated. The one-page questionnaire included demographic characteristics, admission diagnosis, self-reported health status, and asset ownership. The survey assessed household ownership of 10 assets: bike, radio, fowl, phone, electricity, mosquito nets, house material, type of toilet, number of rooms for sleeping, and meals eaten per day. These were selected from a previous population-based study of 1,205 women in the same region completed in July of 2007, the details of which are described elsewhere [20].

Two health workers from each health center were trained to administer the survey. Following the September 2008 data collection period, one trained interviewer from Bitale was transferred to another health center and the other trained interviewer left the post for personal reasons. The two replacement health workers were trained by the project manager and completed interviews in January 2009 and May 2009.

All patients who were admitted to the four health centers were eligible to participate after providing written consent. The parents/guardians of inpatients under the age of 18 provided consent on their children's behalf. If patients were severely ill on admission, study health workers were instructed to interview them only after their conditions stabilized. Patient interviews lasted for approximately 5-10 minutes. Written consent was obtained from all participants. The study received ethical clearance from the Tanzania National Institute for Medical Research and the University of Michigan Institutional Review Board.

### Statistical analysis

We calculated univariate statistics for health center characteristics and demographic variables for all admissions, as well as three admission sub-types: pediatric admissions, obstetric admissions, and other admissions. Individuals under the age of 5 were classified as pediatric admissions. Individuals admitted for deliveries, post-delivery complications, or post-abortion complications were classified as obstetric admissions. Marital status was assessed for adult inpatients. Previous schooling was only assessed for inpatients at least 7 years old.

Inpatients were categorized into wealth groups (quintiles) based on their asset index using population quintile cut-offs in the Kruk et al population-based survey [20]. Asset indices are frequently used to estimate permanent wealth in non-cash economies [21]. Household assets were assigned numeric values and an index was created using principal component analysis. The first component was used to determine asset weights, which were then used to calculate a continuous index of wealth [21-23]. Based on the value for the asset index, households were divided into five wealth quintiles (quintile 1 was designated as poorest and quintile 5 the richest). Individuals missing more than one asset response were not included in the wealth analysis. Assets were imputed for individuals with only one asset response missing, using logit imputation for the dichotomous assets and mean imputation for the number of mosquito nets and daily meals. A bivariate analysis comparing patients excluded from equity analysis to those classified by wealth quintile was completed, showing no meaningful differences between the two groups on demographic and illness factors.

Concentration curves were constructed and concentration indices were calculated for all inpatients and the three admission sub-types. Concentration curves indicate the equity of distribution of a service graphically. Concentration curves have ascending wealth on the x-axis and a health variable on the y-axis, with a 45-degree line indicating equitable distribution and values below this line indicating disproportionate concentration of the variable among the rich. Concentration indices were also calculated for the following subgroups: patients with fee exempt status and patients required to pay a fee. The concentration index is a quantitative measure of the deviation of the concentration curve from the line of equality (45 degrees) and has been widely used in international research to quantify the degree of income inequality [24-27]. A concentration index of zero indicates perfect equity. Since admissions are a health good, concentration curves falling below the line of equity indicate a system that disproportionately benefits the wealthier individuals--i.e., where admissions are more frequent for the wealthy. A larger concentration index indicates greater inequity.

A Wilcoxon rank-sum test was completed comparing the wealth distribution of all inpatients, as well as the defined subgroups, to the wealth distribution of the community population. The same test was performed to compare the wealth distribution of the subgroups to wealth distribution of all inpatients.

## Results

Table 1 summarizes the characteristics of the four health centers. Over the four month period, the mean number of health workers at each clinic ranged from 1.5 to 2.8 for assistant medical officers and clinical officers, mid-level health providers who can engage in limited diagnostics and prescribing, and from 2.5 to 6.3 for nurses and midwives. No physicians were on staff for any of the health centers. The mean number of beds over this period ranged from 22.8 to 39.8.

**Table 1:** Characteristics of four rural health centers in Kigoma, Tanzania over four months^1^

	Bitale	Kakonko	Mabamba	Nguruka
Number of health workers ^2^				
physicians	0	0	0	0
assistant medical officers and clinical officers ^3^	2.3	1.5	1.8	2.8
nurses and midwives	3.3	2.5	3.3	6.3
Beds ^2^	22.8	39.8	36.5	24.5

^1 ^Data was collected in May 2008, September 2008, January 2009, and May 2009.

^2 ^The health workers and the number of beds are averaged over the four month period.

^3 ^Assistant medical officers and clinical officers are mid-level health providers who can engage in limited diagnostics and prescribing

In May 2008, September 2008, January 2009, and May 2009, 2,767 patients were admitted to the four health centers. 2,578, or 93.2%, of inpatients participated in the study. The number of participants recruited from Kakonko, Bitale, Mabamba, and Nguruka are 976, 565, 554, and 483, respectively.

Table 2 describes the characteristics of all admissions and the following sub groups: pediatric admissions, obstetric admissions, and other admissions. One health center worker from Bitale recorded the age of the parent or guardian instead of the age of the child when completing the interview on the child's behalf. As a result, the data from Bitale was not included in the subgroup analysis. For the population as a whole, 24.2% of inpatients were under the age of 5 and 84.2% of inpatients were under the age of 36. The majority of inpatients (78.7%) were female. Of the inpatients older than the legal age of marriage--15 for females and 18 for males--57.8% were currently married. Of the inpatients 7 years and older, 25.2% of inpatients had not attended any school. Eighty-one percent of inpatients described their health status as good or very good. The majority of patients walked to the clinic (50.3%). The most common reasons for admission were malaria (39.3%), delivery (32.0%), respiratory infection (9.7%), and diarrhea or dysentery (5.2%).

**Table 2:** Characteristics of inpatients at four rural health centers in Kigoma, Tanzania over a four month period^1 ^(n = 2578 ^2^)

	All admissions (n = 2578)		Pediatric admissions ^3^,* (n = 485)		Obstetric admissions ^4 ^(n = 823)		Other adult admissions* (n = 806)		Fee-exempt admissions* (n = 1241)	
	**n**	**(%)**	**N**	**(%)**	**n**	**(%)**	**n**	**(%)**	**n**	**(%)**

Age										

< 5	485*	(24.2)	485	(100.0)	0	(0.0)	0	(0.0)	485	(39.2)

5-17	149*	(7.4)	0	(0.0)	39	(4.8)	116	(14.5)	34	(2.8)

18-25	591*	(29.5)	0	(0.0)	399	(48.7)	238	(29.8)	351	(28.4)

26-35	462*	(23.1)	0	(0.0)	296	(36.1)	208	(26.0)	249	(20.1)

> 35	317*	(15.8)	0	(0.0)	85	(10.4)	238	(29.8)	119	(9.6)

Female	2023	(78.7)	266	(55.0)	823	(100.0)	585	(72.8)	1000	(80.7)

Currently married ^5^	828*	(57.8)	0	(0.0)	499	(61.3)	402	(55.8)	447	(59.9)

No schooling ^6^	370*	(25.2)	0	(0.0)	174	(21.9)	209	(27.3)	187	(25.4)

Very good or good health status	2078	(81.0)	402	(84.3)	717	(87.4)	819	(76.3)	1044	(84.9)

Transportation to clinic										

walked	1292	(50.3)	251	(51.9)	411	(50.0)	282	(35.2)	585	(47.2)

biked	810	(31.5)	158	(32.6)	254	(30.9)	317	(39.6)	423	(34.1)

Reason for admission										

Malaria	985	(39.3)	280	(60.0)	0	(0.0)	415	(54.3)	292	(23.7)

Delivery	802	(32.0)	0	(0.0)	773	(93.9)	0	(0.0)	4	(0.3)

Respiratory infection	242	(9.7)	93	(19.9)	0	(0.0)	101	(13.2)	106	(8.6)

Diarrhea/dysentery	130	(5.2)	57	(12.2)	0	(0.0)	24	(3.1)	57	(4.6)

Accident/injury	82	(3.3)	5	(1.1)	0	(0.0)	70	(9.2)	6	(0.5)

Acute abdominal pain	61	(2.4)	2	(0.4)	0	(0.0)	45	(5.9)	3	(0.2)

Abortion	24	(1)	0	(0.0)	24	(2.9)	0	(0.0)	21	(1.7)

Anemia	28	(1.1)	9	(1.9)	0	(0.0)	15	(2)	9	(0.7)

Post-delivery complication	27	(1.1)	1	(0.2)	26	(3.2)	0	(0.0)	7	(0.6)

Poison/snake bite	13	(0.5)	3	(0.6)	0	(0.0)	8	(1.1)	3	(0.2)

Hypertension	8	(0.3)	0	(0.0)	0	(0.0)	6	(0.8)	1	(0.1)

Fever of unknown origin	8	(0.3)	2	(0.4)	0	(0.0)	5	(0.7)	3	(0.2)

Measles	12	(0.5)	0	(0.0)	0	(0.0)	12	(1.6)	0	(0.0)

HIV/AIDS	7	(0.3)	0	(0.0)	0	(0.0)	6	(0.8)	679	(55.2)

Tuberculosis	4	(0.2)	0	(0.0)	0	(0.0)	4	(0.5)	23	(1.9)

Asthma	4	(0.2)	0	(0.0)	0	(0.0)	4	(0.5)	0	(0.0)

Urinary tract infection	4	(0.2)	2	(0.4)	0	(0.0)	0	(0.0)	2	(0.16)

Other	68	(2.7)	13	(2.7)	0	(0.0)	49	(6.4)	15	(1.22)

*Data for health center Bitale was omitted because one interviewer recorded the age of the parent instead of the child for pediatric admissions

^1 ^Data was collected in May 2008, September 2008, January 2009, and May 2009.

^2 ^Totals may not equal 2578 due to missing values.

^3 ^Inpatients classified as pediatric admissions were under the age of 5.

^4 ^Inpatients classified as obstetric admissions were admitted for delivery, post-delivery complications, or post-abortion complications.

^5 ^Marital status was determined for females 15 years and older and for males 18 years and older.

^6 ^No schooling was determined for patients 7 years and older.

**Figure 1:**
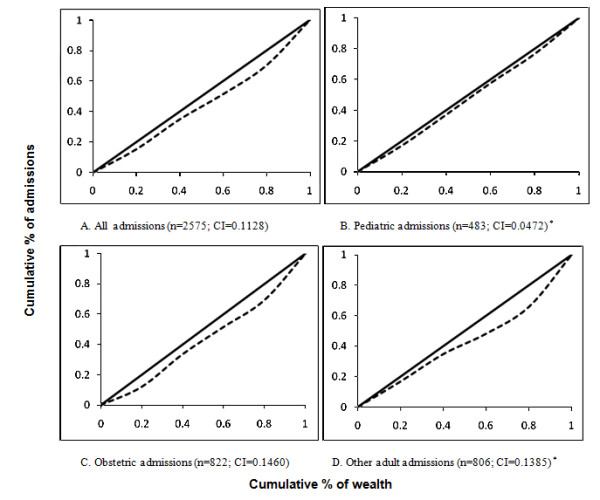
**Concentration curves by wealth quintile for inpatients at four rural health centers in Kigoma, Tanzania over three months^1 ^(n = 2575)**. ^1 ^Data was collected in May 2008, September 2008, January 2009, and May 2009. *Data for health center Bitale was omitted because one interviewer recorded the age of the parent instead of the child for pediatric admissions.

Table 3 describes the wealth distribution and concentration index of all admissions and the aforementioned subgroups. For all inpatients, 15.3% were characterized as the most poor, 19.6% were characterized as very poor, 16.5% were characterized as poor, 18.9% were characterized as less poor, and 29.7% were characterized as the least poor. Figure 1 displays the concentration curves for these groups. For all admissions and for each subgroup the concentration curve falls below the forty-five degree line, indicating wealth-based inequality. The concentration index for all admissions was 0.1128. The concentration indices for the pediatric, obstetric, and other admissions were 0.0472, 0.1460, and 0.1385, respectively.

**Table 3:** Wealth distribution of inpatients at four rural health centers in Kigoma, Tanzania over three months^1 ^(n = 2575 ^2^)

	Community population (n = 1205)		All inpatients (n = 2575)		Pediatric inpatients ^3^, * (n = 483)		Obstetric inpatients ^4 ^(n = 1002)		Other adult inpatients* (n = 806)		Fee-exempt inpatients* (n = 1238)	
	**N**	**(%)**	**n**	**(%)**	**n**	**(%)**	**n**	**(%)**	**n**	**(%)**	**n**	**(%)**
most poor	236	(19.6)	393	(15.3)	82	(17.0)	95	(11.6)	135	(16.8)	181	(14.6)
very poor	246	(20.4)	505	(19.6)	97	(20.1)	182	(22.1)	145	(18.0)	266	(21.5)
poor	236	(19.6)	425	(16.5)	99	(20.5)	129	(15.7)	108	(13.4)	217	(17.5)
less poor	246	(20.4)	487	(18.9)	92	(19.1)	160	(19.5)	142	(17.6)	238	(19.2)
least poor	241	(20.0)	765	(29.7)	113	(23.4)	256	(31.1)	276	(34.2)	336	(27.1)

Concentration index			0.1128		0.0472		0.1460		0.1385		0.0911	
Chi-square of lowest two quintiles to highest two quintiles			< 0.0001		0.0874		< 0.0001		< 0.0001		< 0.0001	
Wilcoxon rank sum, community population			< 0.0001		0.2242		< 0.0001		< 0.0001		0.0001	

*Data for health center Bitale was omitted because one interviewer recorded the age of the parent instead of the child for pediatric admissions

^1 ^Data was collected in May 2008, September 2008, January 2009, and May 2009.

^2 ^Inpatients with more than one asset response missing were excluded from this analysis. For those with only one asset missing, the missing asset was imputed as described in methods, above.

^3 ^Inpatients classified as pediatric inpatients were under the age of 5 and were not admitted for obstetric conditions.

^4 ^Inpatients classified as obstetric inpatients were admitted for delivery, post-delivery complications, or post-abortion complications.

When comparing the ordinal wealth quintiles using Wilcoxon rank sum tests, the wealth distribution of all inpatients (p < 0.0001), obstetric inpatients (p < 0.0001), other inpatients (p < 0.0001), and fee-exempt inpatients (p < 0.001) were significantly different than the wealth distribution in the community population. The wealth distribution of pediatric inpatients (p = 0.2242) did not significantly differ from the population at large. The analyses with continuous asset index had similar results.

To address potential misclassification between single quintiles, we analyzed admissions for the poorest 40% of the population (grouping together quintiles 1 and 2) and the richest 40% (grouping together quintiles 4 and 5) of the population. The richest 40% of the population accounted for 48.6% of admissions whereas the poorest 40% of the population accounted for 35.2% of admissions.

## Discussion

This study assessed the wealth distribution of inpatients admitted to rural health centers in Tanzania to provide insight about equity of inpatient care in Tanzania under current fee exemption policies. We found that in Kigoma Region, Tanzania, wealthier citizens were more likely to use rural health centers for inpatient care than their poorer counterparts. Overall, the richest 20% of the population accounted for 29.7% of admissions to the rural health centers whereas the poorest 20% of the population accounted for only 15.3% of admissions. Admissions were inequitable for obstetric inpatients and other adult inpatients in which the poorest 20% of the population accounted for 11.6% and 16.8% of inpatients, respectively. Inequities were not found for the pediatric inpatients.

The finding that individuals in the wealthiest quintile use health center services at a disproportionately high rate is consistent with other research on inequities [1,28,29]. This study also supports prior research findings that user fee exemptions increase equity but are, by themselves, insufficient to eliminate wealth-based inequities. For fee exempt individuals, the richest 20% of the population accounted for 27.1% of admissions, as compared to 34.6% for patients required to pay a fee.

Informal fees, transportation expenses, or potential lost wages for family members may account for remaining inequities. For example, in a study conducted in the same region of Tanzania, Kruk and colleagues found that, on average, deliveries at a government facility cost women 6268 TZS (approximately 4.20 USD), despite the fee-exempt status for obstetric admissions. Approximately half of delivery costs could be attributed to transportation costs and the remainder to illegal provider fees or fees for supplies [10]. These expenses may not only result in lower utilization by fee-exempt patients, but also result in greater economic hardship for poorer patients as compared to richer patients who use the health centers. Prior research suggests that inpatients in the poorest wealth quintiles may be more likely to reduce expenditures for other goods, such as food, or to borrow or sell items to afford care [30,31].

The relatively larger inequities observed within the obstetric admission group, as compared to the pediatric admission group (both groups fall in the fee exempt category), may indicate that transportation expenses and informal fees are a greater barrier to care for obstetric patients. Fees for drugs and supplies may be particularly high for obstetric admissions since they often involve more complicated procedures and medicines and supplies which may not be routinely available at health centers and thus must be purchased by patients. Similarly, transportation fees for obstetric emergencies may be higher as they may require private transport at night and in bad weather to a greater extent than other admissions. Other factors, such as education and preferences for formal health care, that differ between poorer and richer women, may also limit poor women's demand for facility-based obstetric are [20,32].

This study compared the results of a patient questionnaire to the results of a population-based survey. Each admitted patient completed a 5-10 minute survey with questions on demographics and a 10-question asset index. The high response rate (93.2%) as well as debriefings of the health workers administering the survey confirming that the questionnaire did not interfere with patient care, suggest that this method is feasible for intermittent assessments of equity of inpatient care. While this was done as part of a formal research project, with attendant ethical clearance and scientific review processes, such approaches may be incorporated into ongoing monitoring and evaluation processes in countries. In those instances, routine population-based surveys, such as the Demographic Health Survey or National Census, can be used to determine the asset cutoffs for each quintile and to develop the brief asset tool.

The study has several limitations. There was a two-year interval between the population-based study and the facility study, which may have resulted in some misclassification of wealth groups if asset ownership increased substantially over this time. For example, the cost of mobile phones may have fallen and thus made these more ubiquitous among households [33]. This would lead to an under-estimate of inequities in facility use. The focus of the study was on barriers to access to inpatient services and we did not measure financial hardship due to inpatient care. As noted above, poor families who are able to obtain care frequently face disproportionate financial burdens from the costs of care. In addition, future research should address the role of education in mitigating the effect of poverty on equity of utilization.

## Conclusion

The facility-based study supports prior research on pro-poor health financing and indicated that the current Tanzanian health financing policies may have improved access to health care for children under five, but that additional policies are needed to further close the equity gap, especially for obstetric inpatients. The successful administration of this brief questionnaire in rural Tanzania suggests that this may be a feasible approach to monitoring the effect of health system policies on equitable provision of health care in low-income countries.

## Competing interests

The authors declare that they have no competing interests.

## Authors' contributions

GF participated in data collection, contributed to the analysis, and wrote the first draft of the manuscript. SD helped coordinate data collection, performed the statistical analysis, and contributed to the manuscript. GM assisted in the study design and helped revise the manuscript. LF contributed to analysis and helped revise the manscript. MK concieved of the study, oversaw data collection, and contributed to the manscript. All authors read and approved the final manuscript.
